# Case Report of *Granulicatella adiacens* as a Cause of Bacterascites

**DOI:** 10.1155/2015/132317

**Published:** 2015-11-04

**Authors:** Molly C. Cincotta, K. C. Coffey, Shannon N. Moonah, Dushant Uppal, Molly A. Hughes

**Affiliations:** ^1^School of Medicine, University of Virginia, Charlottesville, VA 22908, USA; ^2^Department of Medicine, University of Virginia, Charlottesville, VA 22908, USA; ^3^Division of Infectious Diseases, Department of Medicine, University of Virginia, Charlottesville, VA 22908, USA; ^4^Division of Gastroenterology, Department of Medicine, University of Virginia, Charlottesville, VA 22908, USA

## Abstract

*Granulicatella adiacens* is a Gram-positive coccus, formerly grouped with nutritionally variant *Streptococcus*, often found as commensal bacteria of the human oral cavity, urogenital tract, and gastrointestinal tract. Prior case reports have demonstrated *Granulicatella* spp. as a pathogen that can cause bacteremia and infective endocarditis particularly of prosthetic valves and pacemaker leads. Here, we report on a unique case of *Granulicatella adiacens* bacterascites in a 50-year-old male.

## 1. Introduction

*Granulicatella adiacens *is a Gram-positive coccus that is nonmotile, nonsporulating, catalase-negative, oxidase-negative, and facultatively anaerobic.* G. adiacens *often grows on agar as satellite colonies adjacent to another organism that provides nutrients such as* Staphylococcus aureus *or* Staphylococcus epidermidis* [1, 2].* G. adiacens *colonies are alpha-hemolytic on sheep-blood agar [1]. The organism has been found to be resistant to optochin and susceptible to vancomycin [1]. The genus* Granulicatella *has three species,* G. adiacens*,* G. elegans*, and* G. balaenopterae*, with only the former two having been isolated from human samples [2].* Granulicatella species *are an uncommon cause of infection. To date, there have been no described cases of* Granulicatella adiacens *as a cause of spontaneous bacterial peritonitis or as an isolate of ascites fluid.

## 2. Case Report

### 2.1. History

A 50-year-old man presented to the emergency department (ED) complaining of symptomatic large volume ascites. The patient and his ascites had been followed by his PCP, who had been adjusting diuretics. However, the patient was failing diuretic management and so was referred to our hospital for a therapeutic paracentesis. The patient's past medical history was significant for a complicated course of gallstone pancreatitis that occurred eight months prior to his current presentation. Chart review revealed several prolonged hospital courses, totaling 53 hospital days, with multiple complications, including necrotizing pancreatitis status after necrosectomy and cholecystectomy, interval placement and subsequent removal of a percutaneous endoscopic gastrostomy tube with jejunal arm (PEG-J) for enteral feeding,* Clostridium difficile* diarrheal infection for which he had received fourteen days of treatment with oral metronidazole, and a new diagnosis of diabetes mellitus. Additionally, he had been found to have new portal vein and splenic vein thromboses during that hospitalization and had since completed six months of therapeutic anticoagulation, originally with Apixaban, which was later switched to Rivaroxaban.

At the time of the patient's current presentation for ascites, most of the above medical problems had resolved. He was tolerating a regular diet and was being followed by his PCP for management of recurrent ascites felt to be due to the above-mentioned portal vein thrombosis.

The patient reported no symptoms other than decreased appetite secondary to bloating and pain in his right groin likely from nonincarcerated hernia. He had no fever, shortness of breath, nausea, vomiting, diarrhea, or significant constipation.

Upon presentation to the ED, computed tomography (CT) of the abdomen showed large volume ascites with no propagation of the portal vein or splenic vein thromboses with interim development of collaterals.

### 2.2. Physical Examination

On exam, the patient's heart rate was normal with a regular rhythm, his lungs were clear, and there was no lower extremity edema. His abdomen was significantly distended, with bulging flanks and a palpable fluid wave. There was a well-healed midline surgical scar with a distal keloid and easily reducible umbilical hernia. Aside from the ascites, the patient had no other sequelae of chronic liver dysfunction; he exhibited no jaundice, no palmar erythema, no spider angiomata, no venous distention, and no asterixis.

### 2.3. Laboratory and Radiological Investigations

In the ED, the patient underwent paracentesis by the gastroenterology (GI) team under normal sterile technique with removal of approximately 4.5 L of clear, yellow fluid. Initial Gram stain of the ascites fluid revealed white blood cells and no bacteria. Pertinent fluid analyses revealed glucose 100 mg/dL, amylase 52 U/L, WBC 372/UL with 48% polymorphonuclear cells (PMNs) and 24% lymphocytes, total protein 4.2 g/dL, and albumin 2.1 g/dL giving a serum albumin ascites gradient of 1.5, consistent with portal hypertension or congestive heart failure. Blood work revealed a peripheral white blood cell count of 6.85 k/*μ*L with a normal differential, platelet count of 326 k/*μ*L, and a lactic acid of 1.3 mmol/L. Ultrasound of the abdomen confirmed that there was no interim propagation of the portal vein thrombosis as well as presence of patent collaterals.

### 2.4. Hospital Course

The patient was discharged from the ED with a follow-up outpatient appointment with a GI physician for further evaluation and management of recurrent ascites. The following day, the GI team was contacted by the Clinical Microbiology Laboratory that Gram-positive cocci in chains were growing in the ascites sample. The GI team contacted the patient's wife who reported that the patient had experienced no clinical status change since the procedure, specifically no fevers or abdominal pain.

The GI team felt that the laboratory result likely represented a contaminant in the sample, and they decided to await speciation before asking the patient to return to the hospital. The following day, the Clinical Microbiology Laboratory reported* Granulicatella adiacens* (not a common laboratory contaminant), so the patient was asked to return to the hospital to be admitted for further monitoring and evaluation.

Upon return to the hospital, the patient reported interval improvement in his abdominal bloating and appetite, and he denied any new symptoms, including fever, abdominal pain, nausea, or diarrhea. His exam was unchanged from prior exam aside from a decrease in his abdominal distention. The GI team performed a diagnostic paracentesis, which revealed glucose of 135 mg/dL, WBC of 813/UL with 72% lymphocytes, 1% PMNs, and 2% atypical lymphocytes, total protein of 4.3 g/dL, and albumin of 2.1 g/dL. Ascites fluid again showed 3+ white blood cells and the bacterial culture again grew* Granulicatella adiacens*. Peripheral blood cultures from two separate peripheral sites were collected and showed no growth after 72 hours. Blood work was not significantly changed from the first presentation.

The patient underwent a transthoracic echocardiogram that showed no evidence of structural cardiac defect and no evidence of endocarditis. His previous CT was reexamined and a large fluid collection was seen with a possible rim of residual pancreatic tissue. Based on this, a magnetic resonance cholangiopancreatography (MRCP) was performed that revealed a fluid collection with possible communication to the left colon. The GI team consulted the general surgery team, who, on review of the imaging and patient status, felt that the risks associated with any surgical intervention outweighed the potential benefits of source control.

Due to the patient having a penicillin allergy, he received intravenous vancomycin for his course of therapy. The five-day antibiotic course was initially complicated by an infusion-related “red man” reaction requiring pretreatment with diphenhydramine and slower infusion times. On hospital day 3, a peripherally inserted central venous catheter (PICC) line was placed, and the patient was discharged home in stable condition. The patient underwent colonoscopy one month later and was found to have three hyperplastic polyps without evidence of malignancy. There was no apparent communication between the colon and the fluid pocket that had been visualized on MRCP. No further blood or ascites cultures were collected in the immediate period after his hospitalization.

## 3. Discussion

*Granulicatella adiacens* is an uncommon cause of infection, but when present, it is most commonly found in the bloodstream. Review of the English literature shows that bacteremia is usually related to device/graft infection or endocarditis (Table 1). Cargill et al. described 17 cases of* Granulicatella* spp. endocarditis between 1997 and 2012 including infections of prosthetic valves and a pacemaker lead [2]. It is suspected that cases of* Granulicatella* spp. infective endocarditis are underreported and that some cases may be a cause of reported culture-negative endocarditis. Bacteremia with* Granulicatella* spp.without endocarditis has been reported, including a single case of infection of aortic atheroma with associated dissection [2]. Other infections have included seeding by prosthetic material or surgery with isolates from brain abscess, CSF, joint space, vertebrae, and breast implant [2].

To our knowledge, we are the first to report a case of* G. adiacens *as a cause of monomicrobial nonneutrocytic bacterascites. Bacterascites is thought to represent colonization of ascites fluid and may progress to the life threatening complication of spontaneous bacterial peritonitis (SBP). The pathogens related to bacterascites are similar to SBP; however the diagnosis of SBP requires the presence of >/= 250 PMN cells/mm^3^ in addition to a positive Gram stain and/or culture of the ascitic fluid [24, 25]. The pathogenesis of SBP is thought to be directly related to hepatic dysfunction, with portal hypertension and local intestinal immunodeficiency leading to intestinal bacterial overgrowth and intestinal mucosal edema leading to breakdown of the epithelial barrier and increased permeability, which can allow for bacterial translocation to other tissues. In patients with ascites fluid that contains protein levels <1 g/dL and/or C3 levels < 13 mg/dL, there is a predisposition for infection [24].

Notably, Altay et al. described a case of* G. adiacens *bacterial peritonitis in a 55-year-old woman, but this was thought to be a peritoneal dialysis-related infection [13]. In that case, the patient was being treated empirically with intraperitoneal cefazolin and gentamicin when repeat cultures of the dialysate grew a single colony of* G. adiacens *susceptible to the empiric regimen. The patient completed 14 days of antibiotic therapy and showed clinical improvement after 2 days with concomitant decrease in ascites leukocytosis [13].

The origin of* G. adiacens *in our patient remains unclear. The possibility of an occult colon cancer leading to bacterial translocation, akin to* Streptococcus bovis*, was considered, but as colonoscopy was unrevealing for malignancy, this is unlikely. As discussed above, CT and MRCP of the abdomen demonstrated a fluid collection near the tail of the pancreas that may have communicated with the wall of the descending colon. Although subsequent colonoscopy did not show evidence of a direct communication, such an area of weakness in the gut wall may have been susceptible to earlier bacterial translocation.

*Granulicatella* spp., when not seen as a causative agent in infective endocarditis or other endovascular infections or bacteremia, have been associated with surgical intervention or seeding from a prosthesis [2]. As this patient had prior extensive abdominal surgery, it is possible that the original introduction of* G. adiacens *occurred during surgery and simply went undetected due to his lack of symptoms. This possibility seems less likely given that the patient had undergone one previous paracentesis several months earlier that grew no organism; however* G. adiacens *is not easily detected and has been found in peritoneal fluid previously documented as culture-negative [13]. It remains questionable whether the presence of* Granulicatella *spp. bacterascites was clinically significant, given the patient's failure to develop peritonitis.

The question of asymptomatic bacterascites is controversial as to whether it may be an incidental finding in patients without laboratory diagnosis of spontaneous bacterial peritonitis. The tendency is to err on the side of treatment in these cases. Older data show that, in patients with nonneutrocytic monomicrobial bacterascites, 62% (65/105) became sterile without antimicrobial intervention. The remaining 38% progressed to SBP [24]. The current recommendations for antibiotic treatment of and prophylaxis against SBP are targeted toward patients with cirrhosis and low ascites protein levels, neither of which describes this patient [25]. With respect to* Granulicatella* spp. as a causative agent, more data would be necessary to draw conclusions regarding the pathogenicity of this particular organism in ascites fluid. Limitations of earlier data are subject to the nutritionally variable nature of this organism, as its prior eponym suggests. Fortunately, 16s rRNA sequencing and mass spectrometry are becoming increasingly more common diagnostic tools, in some cases replacing traditional culture, which often confused this organism with poorly growing* Streptococcus*.

Our patient was treated with a 5-day course of intravenous vancomycin although* Granulicatella adiacens* infections have most commonly been treated with a beta-lactam antibiotic (Table 1). Intravenous vancomycin was chosen in this case because the patient had a penicillin allergy. Additionally, antibiotic susceptibility testing could not be performed for this isolate so beta-lactam antibiotic susceptibility or resistance could not be assessed. A limitation of our report is that successful eradication was not definitively demonstrated, as repeat paracentesis was not performed because the patient was doing well clinically, and our patient was asymptomatic prior to initiation of microbial therapy. Contamination is not suspected, as the organism grew from two separate cultures, obtained two days apart. If our patient manifests symptoms or if future blood or ascites fluid cultures grow* Granulicatella* spp., further evaluation is warranted with blood cultures and a repeat echocardiogram as well as additional abdominal/pelvis imaging. Repeat paracentesis with positive culture could indicate a complication such as an abscess formation or continued reinfection via translocation. At the current time, the patient has been out of the hospital for more than six months, and he has experienced no apparent complications.

This case presents an interesting finding of* Granulicatella *spp. as an infectious agent involved in bacterascites with potential to progress to spontaneous bacterial peritonitis that has not been previously described in the literature. Clinical suspicion and testing for* Granulicatella* spp. in peritoneal fluid may help us to learn more about this commensal organism of the GI tract and its pathogenicity and response to therapy. We would recommend serial paracenteses with monitoring for clearance of the organism from the fluid and/or development of laboratory confirmed neutrocytic SBP for future cases prior to initiation of therapy.

## Figures and Tables

**Table 1:** Clinical characteristics of other cases of *Granulicatella adiacens* reported in the English literature in PubMed.

Age (years)/gender	Site of infection	Antibiotic treatment	Treatment duration (weeks)	Outcome	Reference
58/M	Mitral valve	Vancomycin/gentamicin	NS	Survived	[2]
63/M	Mitral and aortic valves	Amoxicillin/gentamicin	NS	Survived	[2]
38/F	Tricuspid valve	Amoxicillin/gentamicin	NS	Survived	[2]
63/F	Mitral and tricuspid valves	Amoxicillin/gentamicin	NS	Deceased	[2]
71/F	Aortic valve	Penicillin/gentamicin	6	Survived	[2]
31/M	Mitral valve	Oxacillin/gentamicin	NS	Survived	[3]
18/M	Pulmonic valve	Vancomycin/gentamicin/rifampin	6	Survived	[4]
18/F	Mitral valve	Penicillin/gentamicin	NS	Survived	[5]
61/M	Mitral and tricuspid valves	Penicillin/gentamicin	NS	Survived	[5]
30/M	Mitral valve	Penicillin/gentamicin/ceftriaxone	NS	Survived	[5]
28/F	Mitral and aortic valves	Penicillin/gentamicin/vancomycin/teicoplanin	NS	Survived	[5]
57/M	Endocarditis, valve unknown	Ampicillin/gentamicin	2	Survived	[6]
77/F	Aortic valve	Vancomycin/ampicillin/gentamicin	NS	Survived	[2]
68/M	Pacemaker/vertebral osteomyelitis	Penicillin/gentamicin/rifampin	NS	Survived	[7]
41/M	Mitral and aortic valve	Penicillin/gentamicin	NS	Survived	[2]
85/M	Endocarditis, valve not reported	NS	NS	Deceased	[2]
71/M	Mitral valve	Ampicillin/gentamicin	5	Survived	[8]
48/F	Mitral valve	Ceftriaxone/gentamicin	4	Survived	[9]
50/M	Mitral and aortic valve	Penicillin/gentamicin	6	Survived	[10]
63/M	Mitral valve	Ampicillin/gentamicin	4	Survived	[11]
0^*∗*^/M	Sepsis	Vancomycin	2	Survived	[12]
NS	Abdominal aortic graft	NS	NS	Deceased	[2]
NS	Hemodialysis catheter	Vancomycin	NS	Survived	[2]
55/F	Peritonitis	Cefazolin/gentamicin	2	Survived	[13]
32/F	Meningitis postsurgical	Penicillin/gentamicin	4/2	Survived	[14]
53/F	Meningitis postsurgical	Penicillin/gentamicin	4	Survived	[14]
49/F	Meningitis after myelography	Vancomycin/fosfomycin/cefixime/rifampin	1.5	Survived	[14]
2/F	Brain abscess	Ampicillin/rifampin/gentamicin	4.5/4.5/1	Survived^a^	[14]
46/F	Brain abscess postsurgical	Ceftriaxone/gentamicin	1.5	Survived	[15]
32/F	Breast implant	Rifampin/amoxicillin	4 months	Survived	[16]
73/M	Bacteremia/vertebral osteomyelitis	Penicillin/gentamicin	4 months	Survived	[17]
68/F	Knee joint	Cefazolin/gentamicin	4/2	Survived	[18]
46/F	Chronic dacryocystitis	Cephalexin/erythromycin/tobramycin-dexamethasone drops	5 days	Survived	[19]
57/M	Aortic valve	Benzylpenicillin	6	Survived	[20]
55/M	Prosthetic knee	Amoxicillin/rifampin	3 months	Survived	[21]
56/M	Scapular carbuncle	Amoxicillin-clavulanate	NS	Survived	[22]
89/F	Posttraumatic bacteremia	Cefazolin	NS	Survived	[23]

^*∗*^Neonate; NS: not specified; ^a^with permanent neurological sequelae.
